# Supplementary material for: First Isolation and Identification of Homologous Recombination Events of Porcine Adenovirus from Wild Boar
Source: Viruses. 2022 Oct 29;14(11):2400. doi: 10.3390/v14112400 (PMC9694405; doi:10.3390/v14112400)
Supplement: Supplementary file 1 [file viruses-14-02400-s001.zip › Oba et al. Suppl FigS1_recombination_breakpoints 20220924.pptx]

## Slide 1
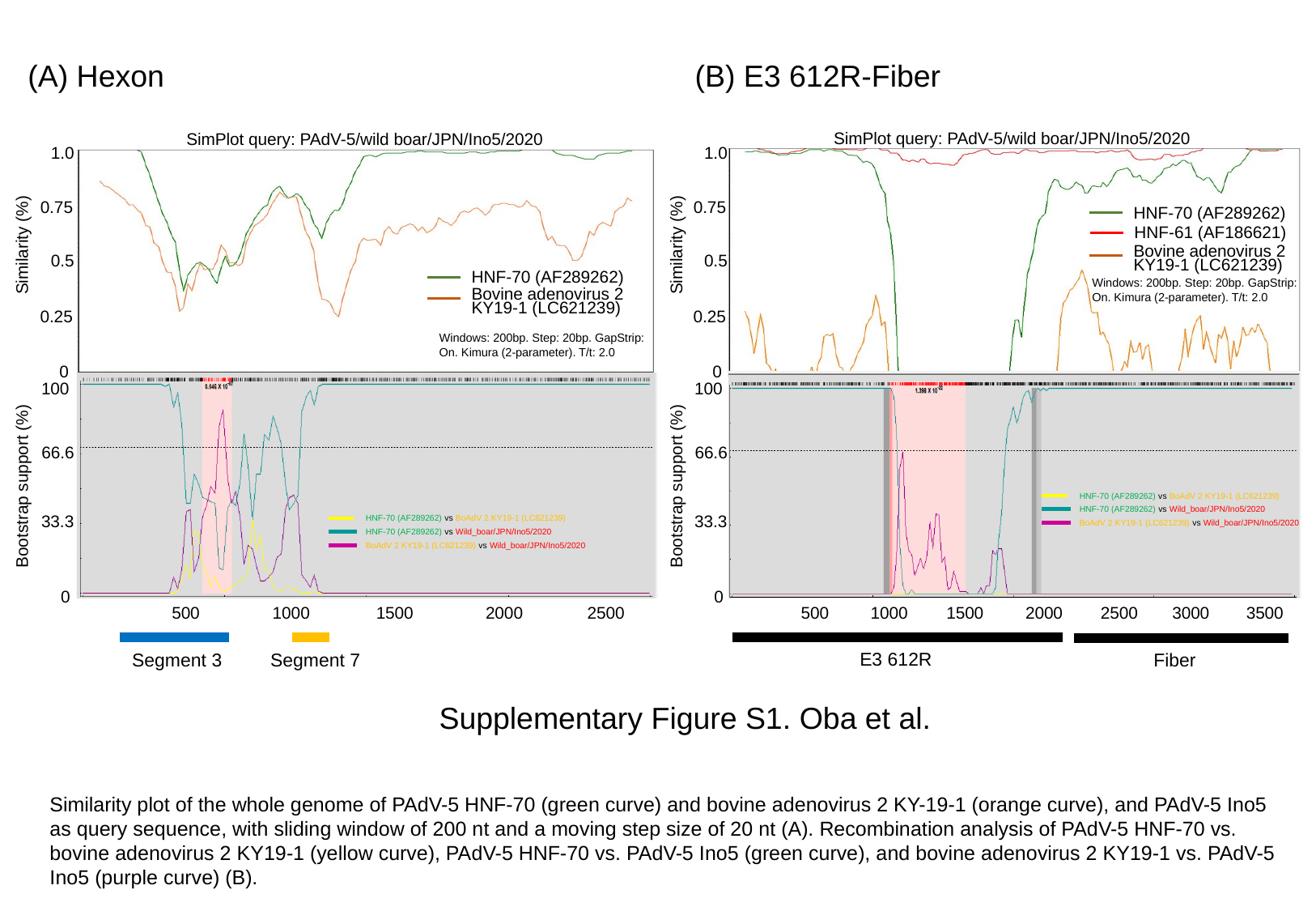

(A) Hexon
(B) E3 612R-Fiber
SimPlot query: PAdV-5/wild boar/JPN/Ino5/2020
SimPlot query: PAdV-5/wild boar/JPN/Ino5/2020
1.0
1.0
0.75
0.75
HNF-70 (AF289262)
HNF-61 (AF186621)
Similarity (%)
Similarity (%)
Bovine adenovirus 2 KY19-1 (LC621239)
0.5
0.5
HNF-70 (AF289262)
Windows: 200bp. Step: 20bp. GapStrip: On. Kimura (2-parameter). T/t: 2.0
Bovine adenovirus 2 KY19-1 (LC621239)
0.25
0.25
Windows: 200bp. Step: 20bp. GapStrip: On. Kimura (2-parameter). T/t: 2.0
0
0
100
100
66.6
66.6
Bootstrap support (%)
Bootstrap support (%)
HNF-70 (AF289262) vs BoAdV 2 KY19-1 (LC621239)
BoAdV 2 KY19-1 (LC621239) vs Wild_boar/JPN/Ino5/2020
HNF-70 (AF289262) vs Wild_boar/JPN/Ino5/2020
33.3
33.3
HNF-70 (AF289262) vs BoAdV 2 KY19-1 (LC621239)
BoAdV 2 KY19-1 (LC621239) vs Wild_boar/JPN/Ino5/2020
HNF-70 (AF289262) vs Wild_boar/JPN/Ino5/2020
0
0
3000
3500
500
1000
1500
2000
2500
500
1000
1500
2000
2500
E3 612R
Segment 3
Segment 7
Fiber
Supplementary Figure S1. Oba et al.
Similarity plot of the whole genome of PAdV-5 HNF-70 (green curve) and bovine adenovirus 2 KY-19-1 (orange curve), and PAdV-5 Ino5 as query sequence, with sliding window of 200 nt and a moving step size of 20 nt (A). Recombination analysis of PAdV-5 HNF-70 vs. bovine adenovirus 2 KY19-1 (yellow curve), PAdV-5 HNF-70 vs. PAdV-5 Ino5 (green curve), and bovine adenovirus 2 KY19-1 vs. PAdV-5 Ino5 (purple curve) (B).
