# Supplementary material for: First Isolation and Identification of Homologous Recombination Events of Porcine Adenovirus from Wild Boar
Source: Viruses. 2022 Oct 29;14(11):2400. doi: 10.3390/v14112400 (PMC9694405; doi:10.3390/v14112400)
Supplement: Supplementary file 1 [file viruses-14-02400-s001.zip › Oba et al. Suppl FigS2_hexon 3D 20220924.pptx]

## Slide 1
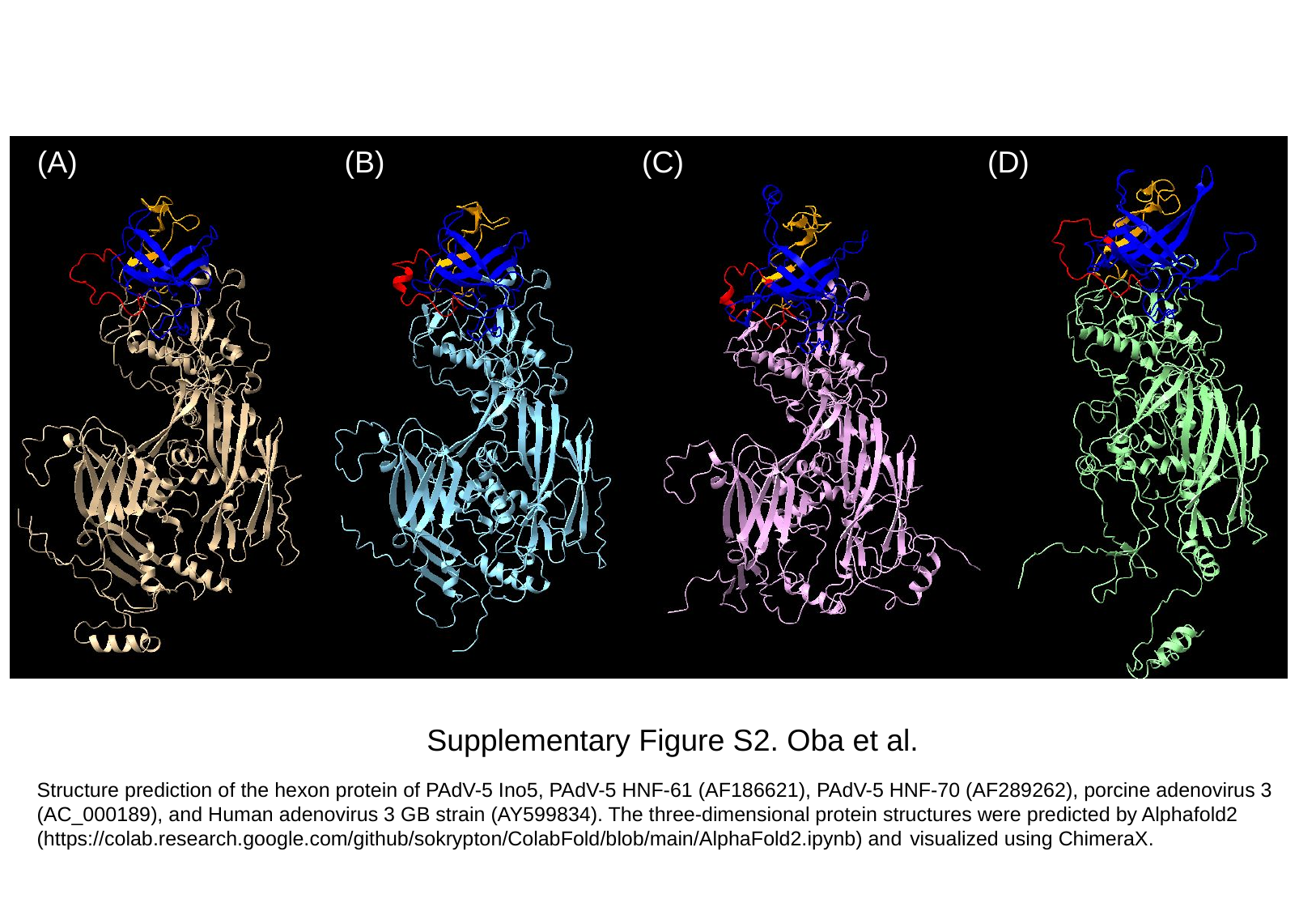

(A)
(B)
(C)
(D)
Supplementary Figure S2. Oba et al.
Structure prediction of the hexon protein of PAdV-5 Ino5, PAdV-5 HNF-61 (AF186621), PAdV-5 HNF-70 (AF289262), porcine adenovirus 3 (AC_000189), and Human adenovirus 3 GB strain (AY599834). The three-dimensional protein structures were predicted by Alphafold2 (https://colab.research.google.com/github/sokrypton/ColabFold/blob/main/AlphaFold2.ipynb) and visualized using ChimeraX.
