# Supplementary material for: First Isolation and Identification of Homologous Recombination Events of Porcine Adenovirus from Wild Boar
Source: Viruses. 2022 Oct 29;14(11):2400. doi: 10.3390/v14112400 (PMC9694405; doi:10.3390/v14112400)
Supplement: Supplementary file 1 [file viruses-14-02400-s001.zip › Oba et al. Suppl FigS3_fiber 3D 20221024.pptx]

## Slide 1
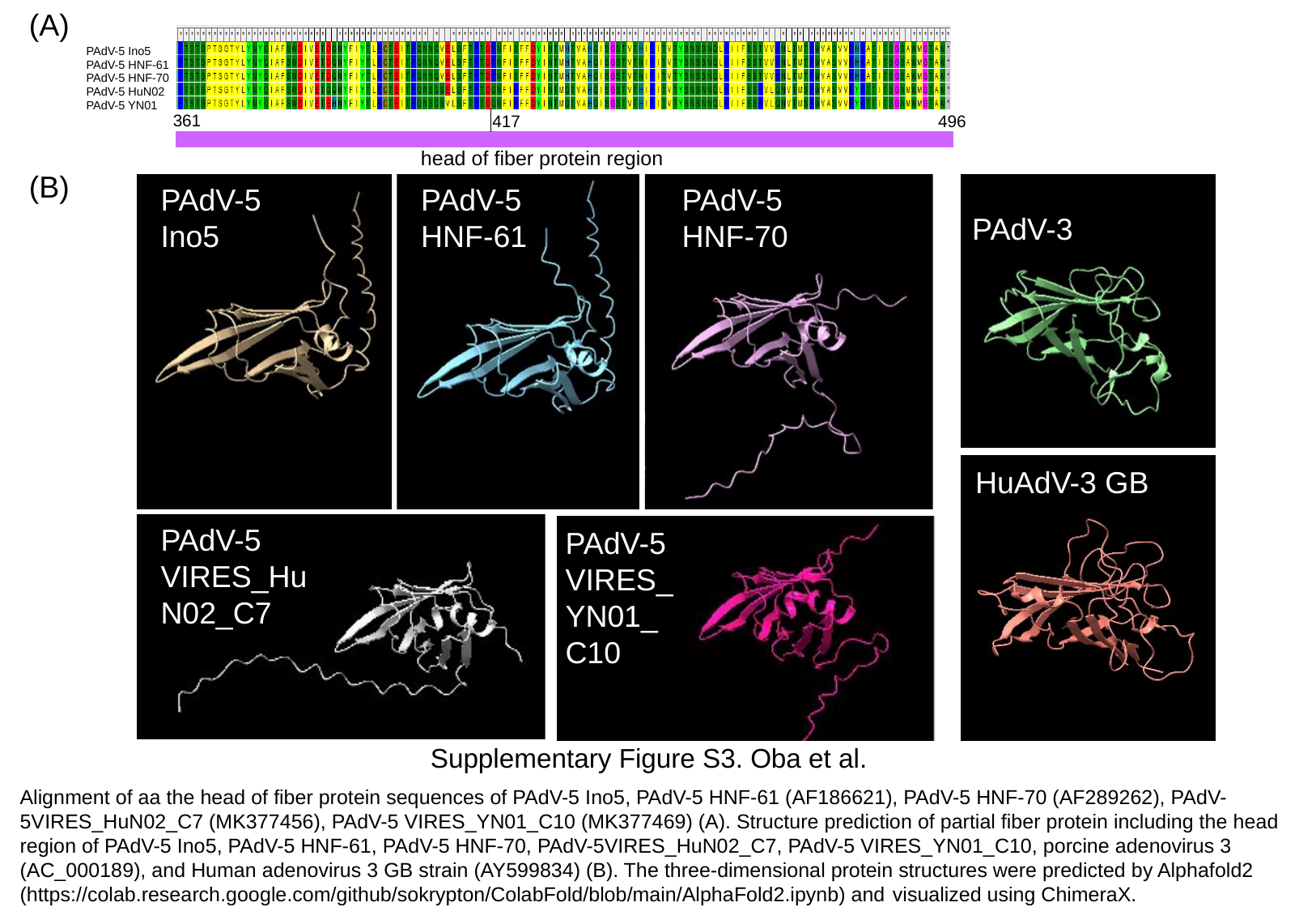

(A)
PAdV-5 Ino5
PAdV-5 HNF-61
PAdV-5 HNF-70
PAdV-5 HuN02
PAdV-5 YN01
361
496
417
head of fiber protein region
(B)
PAdV-5
Ino5
PAdV-5
HNF-61
PAdV-5
HNF-70
PAdV-3
HuAdV-3 GB
PAdV-5
VIRES_HuN02_C7
PAdV-5
VIRES_YN01_C10
Supplementary Figure S3. Oba et al.
Alignment of aa the head of fiber protein sequences of PAdV-5 Ino5, PAdV-5 HNF-61 (AF186621), PAdV-5 HNF-70 (AF289262), PAdV-5VIRES_HuN02_C7 (MK377456), PAdV-5 VIRES_YN01_C10 (MK377469) (A). Structure prediction of partial fiber protein including the head region of PAdV-5 Ino5, PAdV-5 HNF-61, PAdV-5 HNF-70, PAdV-5VIRES_HuN02_C7, PAdV-5 VIRES_YN01_C10, porcine adenovirus 3 (AC_000189), and Human adenovirus 3 GB strain (AY599834) (B). The three-dimensional protein structures were predicted by Alphafold2 (https://colab.research.google.com/github/sokrypton/ColabFold/blob/main/AlphaFold2.ipynb) and visualized using ChimeraX.
