# Supplementary material for: First Isolation and Identification of Homologous Recombination Events of Porcine Adenovirus from Wild Boar
Source: Viruses. 2022 Oct 29;14(11):2400. doi: 10.3390/v14112400 (PMC9694405; doi:10.3390/v14112400)
Supplement: Supplementary file 1 [file viruses-14-02400-s001.zip › Oba et al. Suppl FigS4_Intranuclear inclusion body in the kidney medulla cells 20220924.pptx]

## Slide 1
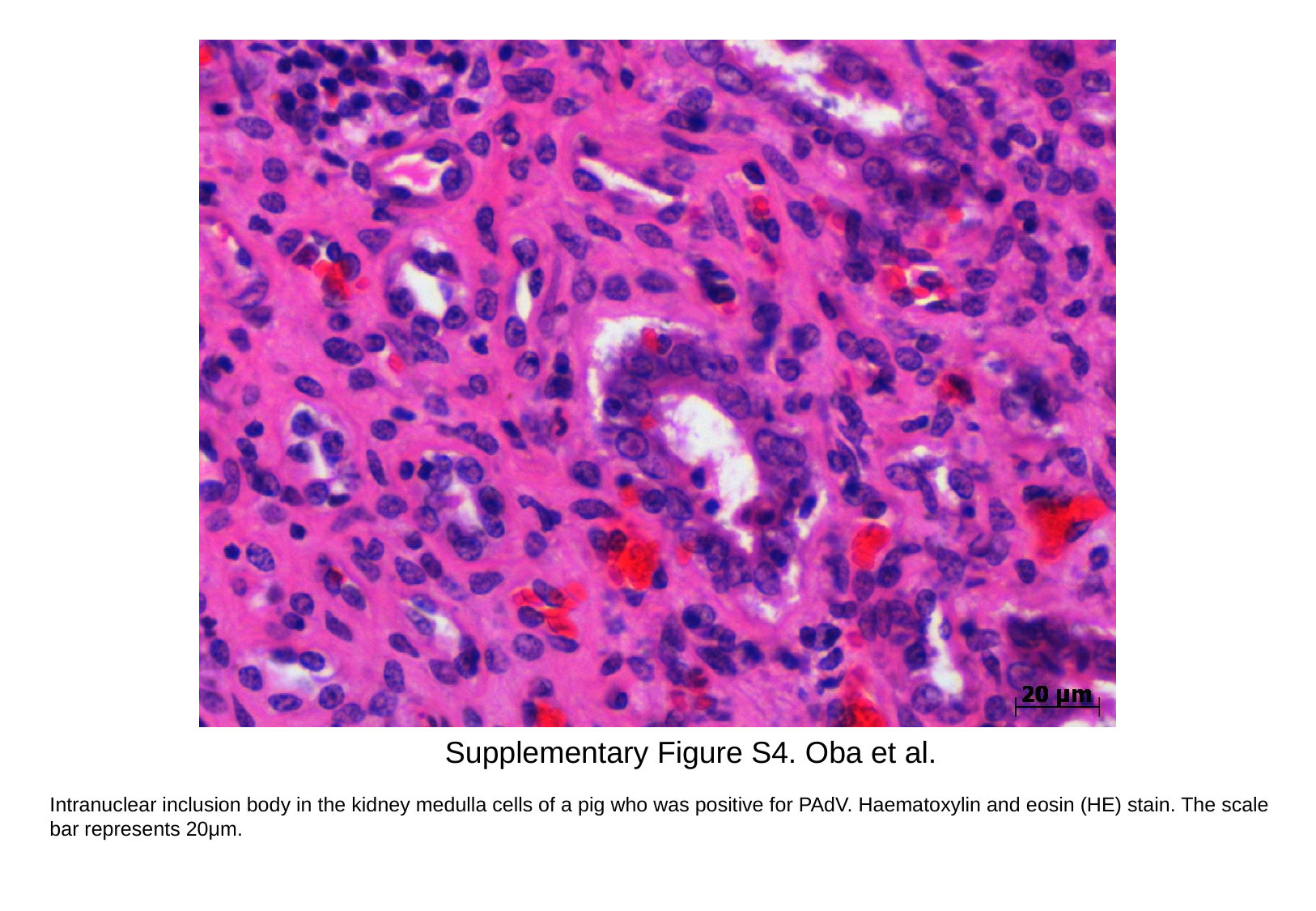

Supplementary Figure S4. Oba et al.
Intranuclear inclusion body in the kidney medulla cells of a pig who was positive for PAdV. Haematoxylin and eosin (HE) stain. The scale bar represents 20μm.
